# Reduced vasodilator function following acute resistance exercise in obese women

**DOI:** 10.3389/fphys.2014.00253

**Published:** 2014-07-07

**Authors:** Nina C. Franklin, Mohamed Ali, Melissa Goslawski, Edward Wang, Shane A. Phillips

**Affiliations:** ^1^Department of Physical Therapy, University of Illinois at ChicagoChicago, IL, USA; ^2^Integrative Physiology Laboratory, College of Applied Health Sciences, University of Illinois at ChicagoChicago, IL, USA; ^3^Department of Biomedical and Health Information Sciences, University of Illinois at ChicagoChicago, IL, USA; ^4^Department of Medicine, University of Illinois at ChicagoChicago, IL, USA

**Keywords:** endothelium, vasodilation, acute exercise, obesity, women

## Abstract

Obesity contributes to stress induced impairments in endothelium-dependent vasodilation (EDV), a precursor to atherosclerosis. Since obesity is associated with inflammation and oxidative stress, we sought to determine if a single bout of strenuous weight lifting (SWL) reduces EDV among sedentary obese adults. Participants included 9 obese (OB) (BMI 30.0–40.0 kg/m^2^) and 8 lean (LN) (BMI 18.5–24.9 kg/m^2^) sedentary young women. All participants underwent a single bout of SWL using a progressive leg-press protocol. Brachial artery flow-mediated dilation (FMD) (an index of EDV) was determined using ultrasonography before and after SWL. Sublingual nitroglycerin (NTG) was used to determine brachial artery endothelium-independent vasodilation following SWL. Brachial artery FMD was significantly reduced in OB and LN women (LN: 6.4 ± 1.6%, *p* = 0.22) after SWL. There was no difference in the magnitude of change pre- and post-SWL between groups (OB: −2.4 ± 0.6% and LN: −2.2 ± 1.6%, *p* = 0.84). Dilation to NTG was lower in OB (21.6 ± 1.3%) compared to LN women (27.6 ± 2.1%, *p* = 0.02) and associated with body weight (*r* = −0.70, *p* = 0.01). These data suggest that EDV is reduced in woman after acute resistance exercise. Dilations to NTG were lower in obese compared to lean woman and associated with body weight suggesting that changes in sensitivity of blood vessels to NO occurs during obesity. These findings may be important for understanding vascular risk following acute exercise in obesity.

## Introduction

Rates of overweight and obesity have reached epidemic proportions (Flegal et al., 2012) with currently 2 in 3 adults either overweight or obese. In fact, atherosclerotic cardiovascular disease (CVD) is the third leading cause of death among women between the ages of 25 and 44 and obesity independently contributes to increased risk in this particular demographic (Mosca et al., 1997). Vascular dysfunction may play a pivotal role in the development of atherosclerosis in obesity (Sturm et al., 2009). Increased body fat is associated with increased reactive oxygen species production (ROS) (Anfossi et al., 2010) and impaired nitric oxide (NO)-mediated endothelium-dependent vasodilation (EDV) (Al et al., 2001). While impaired endothelium-independent dilation (EID), indicative of smooth muscle dysfunction, is also linked to the development of atherosclerosis, most research suggests that it is preserved (Arkin et al., 2008).

Resistance exercise training is recommended for preventing weight gain and reducing the risk of obesity associated cardiovascular risk (Mason et al., 2007) and may reduce the ill effects of chronic stress on cardiovascular function (i.e., psychological stressors) (Paine et al., 2013). A previous study found that resistance exercise training (2 days per week) for 18 weeks had no effect on endothelial function in healthy post-menopausal women (Casey et al., 2007), while others have found improvements in endothelial function after 16 weeks of resistance exercise when combined with other lifestyle interventions of aerobic exercise and calorie reduction (Cotie et al., 2014). Acute resistance exercise increases blood flow intermittently, yields increased shear stress and improves nitric oxide (NO)-mediated vasodilation (Tinken et al., 2010). However, acute resistance exercise is associated with elevated systolic and diastolic blood pressures (Sale et al., 1994) to levels known to impair vascular function (Jurva et al., 2006).

Previous studies on stress-induced cardiovascular dysfunction have focused on the neural and hormonal regulation of cardiovascular function following acute psychological challenges (Chrousos, 2000, 2009). Research on the direct effects of acute physical stressors on vasodilator function and the contribution of obesity to these responses has been limited. In previous studies from our laboratory brachial artery flow mediated dilation (FMD) was reduced following acute weight lifting in sedentary compared to exercise-trained men, despite similar elevations in blood pressure during exercise (Phillips et al., 2011). Resistance exercise is an important component of exercise prescription in overweight and obesity to promote lean body mass and insulin sensitivity (Laskowski, 2012). Therefore, there is a need to determine the clinical impact of different modes of exercise (including resistance exercise) on vascular function in woman since (1) young women suffer a disproportionate burden of morbidity and mortality attributable to obesity and have higher morbidity and mortality rates after myocardial infarction compared to men (Maas et al., 2011) and (2) intense exercise which may be experienced particularly in the initial stages of an exercise program may be harmful to blood vessel function. For example, acute high intensity resistance exercise (>80% 1RM) can reduce brachial FMD (Phillips et al., 2011; Choi et al., 2014). In contrast, moderate intensity, aerobic exercise (50–65% VO_2max_) has been shown to improve FMD in lean individuals (Rooks et al., 2011) or reduce FMD in overweight individuals (Harris et al., 2008). The purpose of this study was to determine whether a single bout of weight lifting reduces FMD in sedentary obese women compared to lean woman in order to determine if obesity (in the absence of other risk factors) translates to more severe reductions in FMD following a strenuous exercise session. In addition, we sought to determine if changes in FMD associated with SWL and are greater in obese compared to lean women. We hypothesized that acute physical stress (in the form of a single session of weight lifting) will reduce vascular function and that this change is augmented in obese compared to sedentary lean women.

## Materials and methods

### Study population

Seventeen women were studied and were recruited in the community. Initially, each participant was screened by telephone and if inclusion criteria were met, they were invited for an in-person screening at which time eligibility was confirmed upon completion of a medical and exercise history questionnaire and physical examination. We included woman who were 18–40 years of age, obese (OB) (BMI 30.0–40.0 kg/m^2^) or lean (LN) (BMI 18.5–24.9 kg/m^2^), and sedentary (less than 150 min of moderate physical activity/week).We excluded individuals with a history of CVD, diabetes mellitus or thyroid dysfunction, woman who were pregnant or recently pregnant and lactating, woman with a history of cancer, history of smoking (for at least 6 months prior to participation), amenorrhea or irregular menses, and use of vasoactive medications. Written informed consent was obtained prior to participation. The study protocol was approved by the Office for the Protection of Research Subjects and the Institutional Review Board of the University of Illinois at Chicago.

### Study design, biochemical, blood pressure, and body composition measurements

**Figure 1:**
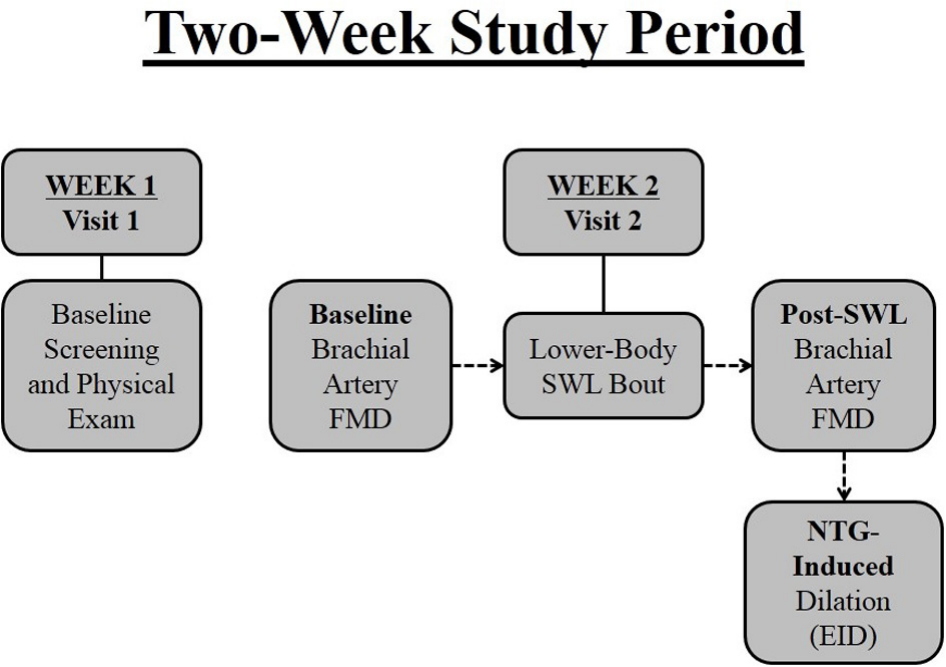
**Two-week research study timeline**. Subjects were screened at baseline (Visit 1) by way of questionnaires and a physical examination. Approximately 1 week later (Visit 2) brachial artery flow-mediated dilation (FMD) was assessed in all subjects prior to and after performance of a single bout of bilateral lower-body strenuous weight lifting (SWL). Subjects were administered nitroglycerin approximately 10 min after FMD for measurement of brachial artery endothelium-independent dilation (EID).

All individuals were evaluated at the University of Illinois at Chicago, Clinical Research Center after a 12-h overnight fast between 0700 and 1100 in a temperature controlled study suite. Figure 1 is a schematic summarizing the study design. Subjects were screened for eligibility at Visit 1. Approximately 1 week later (Visit 2) brachial artery flow-mediated dilation (FMD) was assessed in all subjects prior to and 30 min after performance of a single bout of bilateral lower-body strenuous weight lifting (SWL). Venous blood samples were drawn before and immediately following resistance exercise from an antecubital vein.

Plasma was separated by centrifugation for off-site laboratory analysis of total cholesterol, high-density lipoproteins (HDLs), low-density lipoproteins (LDLs), and glucose (Alverno Clinical Laboratories, LLC; Hammond, IN). Total cholesterol, HDLs, LDLs, and triglycerides were measured using spectrophotometric assays with intra-assay variances of 1.4, 3.0, 2.5, and 2.0%, respectively. Glucose concentration was measured using the glucose oxidase procedure (Beckman Autoanalyser II; Beckman Coulter Inc.; Fullerton, CA) with intra-assay variance of 1.5%. Systolic blood pressure (SBP) and diastolic blood pressure (DBP) were measured after at least 10 min of rest using a standard manual mercury sphygmomanometer with an appropriate cuff size and during the last repetition of each set of exercise. Waist circumference was measured at the narrowest part of the waist (above the umbilicus and below the xiphoid process) (Ohrvall et al., 2000). Body composition was determined by dual energy x-ray absorptiometry (DEXA) using established methods (Mattila et al., 2007).

### Determination of vasodilator function

Brachial artery FMD was used as a measure of endothelium dependent vasodilation (EDV) using techniques previously described (Corretti et al., 2002; Phillips et al., 2011). In premenopausal women, FMD may vary during the menstrual cycle (Hashimoto et al., 1995); therefore, subjects were recruited during the early follicular phase of menses. In the supine state, ultrasound imaging (MicroMaxx; Sonosite; Seattle, WA) of the brachial artery was performed in a longitudinal plane at a site 1–3 cm proximal to the antecubital fossa of the arm (Phillips et al., 2011). Baseline images were recorded and a blood pressure cuff was placed on the forearm and inflated to 50 mmHg above SBP for 5 min. Arterial diameter was determined during peak hyperemia after release of the blood pressure cuff from the forearm. To assess dilation, 10 s of images were captured 1–3 min after cuff release. Flow velocity was recorded at baseline and just after cuff release where maximal velocity was observed. Brachial artery reactivity to FMD was assessed before and 30 min following a single bout of SWL. The original position of the ultrasound probe was marked and measured according to the distance from the antecubital crease and the post-exercise examination was performed in the same position. Ten minutes after recording the last brachial artery diameter measurement following exercise, EID was determined with 0.4 mg of sublingual nitroglycerin (NTG) for 5 min. Since there is a blood pressure lowering effect of NTG making it unsafe to administer immediately prior to maximal exercise, we analyzed NTG dilations in a separate group of age and BMI matched adults who did not perform exercise. All ultrasonographic images were recorded and transferred to a PC for offline analysis of FMD and NTG responses using edge-detection software (Medical Imaging; Iowa City, IA). The coefficient of variation for FMD% was 6.3 and 3.2% for NTG-induced dilation. To estimate brachial artery wall shear stress, peak shear rates (SR) were calculated during FMD using the following equation: peak SR = maximal flow velocity (mm/s) ÷ diameter (mm). FMD was normalized for the peak SR using the following equation: normalized FMD = FMD ÷ peak SR (Phillips et al., 2011).

### Exercise protocol

The exercise protocol involved performance of a single bout of bilateral lower-body weight lifting using a variable-resistance leg press machine (Hoist HD-1610 Selectorized Leg Press; Hoist Fitness Systems; San Diego, CA). After becoming familiarized with the leg press machine, participants performed a warm up period of 1–2 sets of 10 repetitions at a perceived capacity of approximately 30–40% of 1-repetition maximum (RM; calculated with a prediction equation) (Kemmler et al., 2006). Then 3–4 sets of 10 repetitions at a perceived capacity of approximately 80–90% of 1-RM performed with a final isometric hold. A 2-min rest interval was allotted between each set. Exercise heart rate and the 10-point Borg rating of perceived exertion (RPE) scale were used as indices of intensity after each set. However, all volunteers completed a minimum of 4 sets of 10 repetitions or to failure on the final set. The RPE scale was not used as an intensity threshold since (1) the perceived capacity was 80–90% 1RM and (2) the goal was to elicit similar increases in BP between participants (Jurva et al., 2006).

### Statistics

Results are expressed as mean ± standard deviation (SD), unless otherwise stated. Normality of the distribution was confirmed for all data using the Shapiro-Wilk test. Differences in physiological and physical characteristics (lipid panels, glucose measurements, vital signs, and anthropometric measurements) as well as dietary characteristics between OB and LN subjects were compared using student *t*-test. Pre-post differences in hemodynamic and vascular characteristics were compared using paired *t*-test within OB and LN groups. The mixed effects model was performed to examine the effects of acute resistance exercise between OB and LN subjects on hemodynamic and vascular variables including brachial artery diameter, maximum percentage change in diameter (FMD), and normalized FMD. For each outcome variable, group (OB vs. LN), time (pre vs. post) and group^*^time interaction were modeled as fixed effects and subject intercept was modeled as random effect. Using only baseline data, we also conducted linear regression analyses to investigate the association of obesity with brachial artery FMD, independent of potential confounding effects. Pearson's correlations were used to evaluate how brachial artery reactivity to FMD and NTG responses relate to physiological and physical characteristics. Throughout the paper the effect size was computed using Cohen (1988). Cohen's *d* is an appropriate effect size for the comparison between two means. It indicates the standardized difference between two means, and expresses this difference in SD units (Δ mean/SD). Due to its pilot nature, we did not perform a formal power analysis for the current study. With sample size of 8 and 9 in each group, the study will have approximately 80% power to detect a large effect size of 1.5 standard unit mean difference between OB and LN using a two-sided alpha of 0.05. The results of our data could provide a more precise power estimate for future studies. Throughout the paper, the level of statistical significance for all analyses was set at two-sided *P* < 0.05. Data were analyzed using SPSS software (Version 19.0; IBM Corp., Armonk, NY).

## Results

### Baseline and vascular characteristics

Baseline characteristics for the OB (*n* = 9) and LN (*n* = 8) women who underwent a single bout of SWL are presented in Table 1. As was expected, body weight was significantly different between sedentary OB and LN women. In addition, all body weight-related characteristics including BMI, body fat percentage, and waist circumference were significantly different between OB and LN women. Total cholesterol and LDL cholesterol were also higher in OB compared to LN women. Table 2 shows hemodynamic and vascular characteristics before and after SWL. Although the participants were not hypertensive (SBP < 140 mmHg), between-group comparisons showed significantly increased SBP and DBP levels in the OB compared to LN women. In addition, FMD and normalized FMD was reduced after compared to before SWL in OB but not in LN women (Table 2). None of the interactions between group and time for hemodynamic and vascular variables listed in Table 2 were found significant in the mixed effects ANOVA models.

**Table 1:** **Cardio-metabolic characteristics of study participants**.

	**Lean (***n*** = **8**)**	**Obese (***n*** = **9**)**	***P*-value**
Height, cm	164.7 ± 8.2	160.0 ± 7.8	0.238
Weight, kg	58.4 ± 6.5	88.5 ± 16.0^*^	<0.001
BMI, kg/m^2^	21.5 ± 1.4	34.2 ± 3.3^*^	<0.001
Body fat (%)	27.3 ± 7.4	42.8 ± 4.5^*^	<0.001
Waist circumference, cm	71.7 ± 5.1	95.2 ± 7.8^*^	<0.001
Total cholesterol, mmol/l	4.0 ± 0.6	5.0 ± 0.6^*^	0.016
LDL, mmol/l	2.0 ± 0.6	2.8 ± 0.6^*^	0.005
HDL, mmol/l	1.6 ± 0.3	1.4 ± 0.3	0.163
Glucose, mmol/l	4.6 ± 0.6	4.9 ± 0.3	0.375
Maximum SBP, mm/Hg	187.8 ± 25.7	188.5 ± 18.0	0.740
Maximum DBP, mm/Hg	123.5 ± 12.2	114.0 ± 11.1	0.556
Maximum weight lifted, kg	103.6 ± 11.0	107.3 ± 10.2	0.639
Maximum heart rate (bpm)	130.9 ± 8.9	137.4 ± 7.1.	0.09

*All values expressed as mean (± SD)*.

* *P < 0.05, Statistically significant*.

*BMI, body mass index; LDL, low density lipoprotein; HDL, high density lipoprotein; SBP, systolic blood pressure; DBP, diastolic blood pressure*.

**Table 2:** **Hemodynamic and vascular characteristics of participants**.

	**Lean (***n*** = **8**)**				**Obese (***n*** = **9**)**			
	**Before SWL**	**After SWL**	**Cohen's *d***	***P*-value**	**Before SWL**	**After SWL**	**Cohen's *d***	***P*-value**
Resting heart rate, beats/min	65.0 ± 8.5	70.1 ± 9.6	0.56	0.078	69.7 ± 11	70.9 ± 11	0.11	0.790
Systolic BP, mm Hg	103.8 ± 7.4	105.3 ± 10	0.17	0.332	119.9 ± 10	119 ± 8	0.01	0.65
Diastolic BP, mm/Hg	60.6 ± 3.4	63.9 ± 6.2	0.66	0.063	76.6 ± 9	75 ± 13	0.14	0.24
Baseline diameter, mm	3.2 ± 0.3	3.1 ± 0.03	0.47	0.596	3.4 ± 0.3	3.4 ± 0.3	0.30	0.447
Brachial artery FMD (%)	8.5 ± 3.7	6.4 ± 4.5	0.51	0.193	10.7 ± 1.2	8.3 ± 1.8^*^	1.57	0.002
Baseline BFV, cm/s	85.3 ± 15.5	90.5 ± 40	0.17	0.615	64.4 ± 21.3^†^	72.7 ± 26	0.35	0.270
Peak BFV, cm/s	136.7 ± 40	140.7 ± 40	0.10	0.779	135.1 ± 37.8	143.4 ± 43	0.21	0.415
Baseline SR, s^−1^	267.2 ± 47	287.8 ± 112	0.24	0.676	193.9 ± 63	218.8 ± 91	0.32	0.561
Peak SR, s^−1^	385.6 ± 130	421.1 ± 81	0.33	0.468	375.0 ± 123	404.9 ± 189	0.19	0.365
Normalized FMD	0.018 ± 0.011	0.011 ± 0.01	0.67	0.102	0.032 ± 0.009	0.024 ± 0.009^*^	0.89	0.008

*All values expressed as mean (±SD)*.

* *P < 0.05, Statistically significant compared to Before SWL (P < 0.05)*.

*BP, blood pressure; FMD, flow-mediated dilation; SR, shear rate; BFV, blood flow velocity; FMD Δ, absolute change in FMD; NTG, nitroglycerin; ND, not determined*.

### Effects of acute exercise on brachial flow and NTG dilations

**Figure 2:**
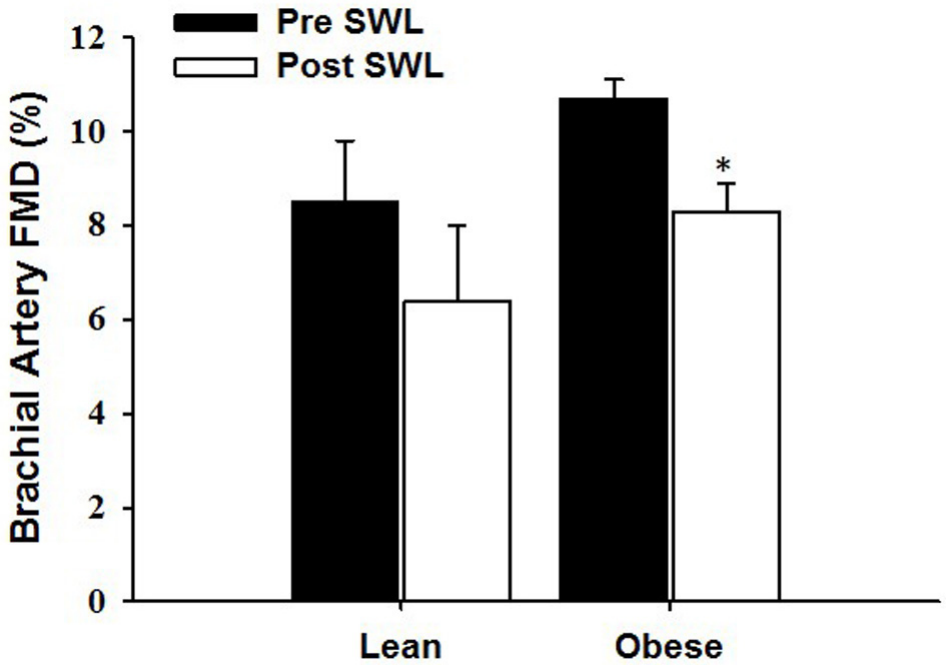
**The effect of a single bout of strenuous weight lifting (SWL) on brachial artery flow-mediated dilation (FMD) in lean (LN) and obese (OB) sedentary women**. ^*^Significant difference observed after SWL vs. before SWL (*p* < 0.05).

**Figure 3:**
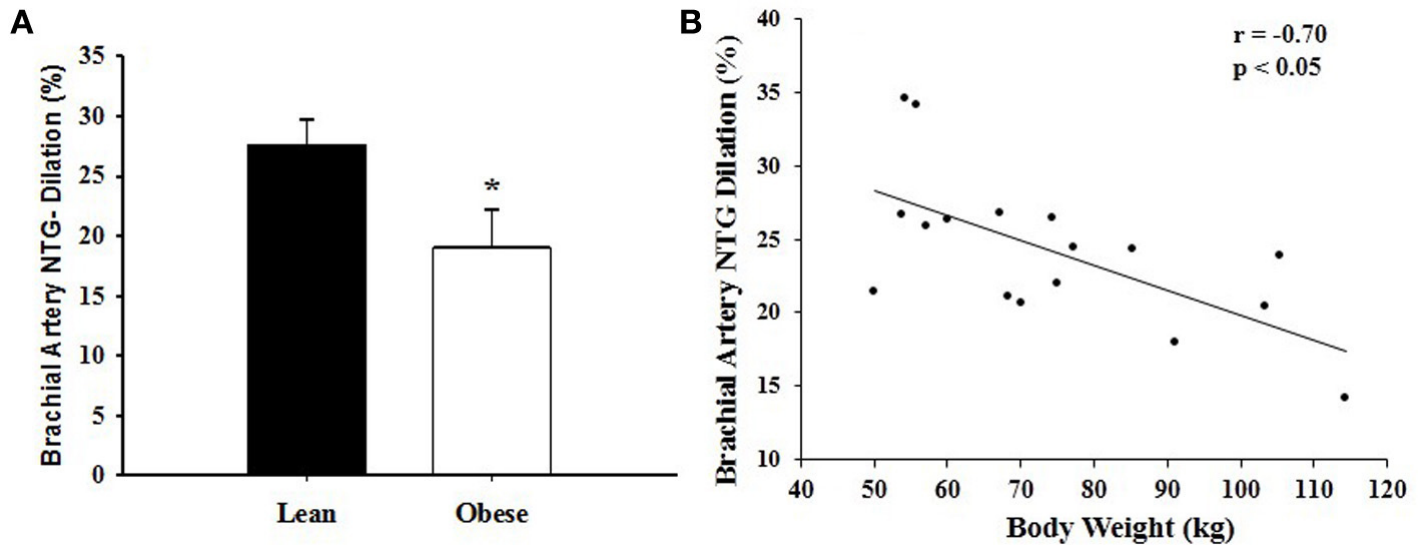
**(A)** Nitroglycerin (NTG)-induced dilation of the brachial artery in lean (LN) and obese (OB) sedentary women after a single bout of strenuous weightlifting (SWL). ^*^Significant difference observed in OB vs. LN (*p* < 0.05). **(B)** Correlation between nitroglycerin (NTG)-induced dilation of the brachial artery and body weight in sedentary women after a single bout of strenuous weight lifting (SWL). There was a negative correlation between brachial artery NTG-induced dilation and body weight (*r* = −0.70, *p* < 0.01).

The RPE during the last repetition was 9.4 ± 0.7 in obese and 9.5 ± 0.8 in lean women. Brachial artery FMD was significantly reduced after SWL in OB group but not in LN group (Figure 2). In the mixed models, however, the Group^*^SWL interaction was not significant, indicating that the slop of change in brachial artery FMD was not difference between LN and OB groups after SWL. There was a significant SWL effect (*b* = −0.024, *p* = 0.038) suggesting that the overall brachial artery FMD was significantly reduced after the SWL exercise (Table 3). NTG-dilation was lower in OB (21.6 ± 1.3%) compared to LN women (27.6 ± 2.1%, *p* = 0.02) (Figure 3A) and this response was negatively correlated with body weight (*r* = −0.70, *p* = 0.01) (Figure 3B), BMI (*r* = 0.62, *p* = 0.02), and WC (*r* = 0.67, *p* = 0.03) but not completely with %BF (*r* = 0.41, *p* = 0.10). Furthermore, correlations were independent of other physiological and physical characteristics. In separate studies (*n* = 12) obese women recruited to perform NTG dilations before exercise had similar NTG dilation (21.9 ± 1.9%) compared to after SWL.

**Table 3:** **Mixed effects model on brachial artery FMD (%)**.

**Effect**	**Coefficient estimate (*b*)**	**Standard error (*SE*)**	***P*-value**
Intercept	0.083	0.010	<0.0001
Group (LN vs. OB)	−0.018	0.014	0.2185
SWL (post vs. pre)	−0.024	0.011	0.0383
Group^*^SWL interaction	0.002	0.015	0.9125

* *Mixed effects model was performed using maximum likelihood method with unstructured covariance*.

## Discussion

The major findings of this study are that: (1) a single bout of strenuous physical stress (induced by weight lifting) reduces EDV in obese women who are sedentary, and (2) nitroglycerin-induced endothelium-independent dilation are reduced in sedentary obese compared to sedentary lean women measured after a single bout of SWL.

### Vascular reactivity and acute exertion in obesity

Under normal physiologic conditions, NO is the chief vasodilator released by the endothelium and functions in modulating smooth muscle tone and inflammation (Vanhoutte et al., 2009). In addition, NO has anti-proliferative effects on the vascular wall and plays a critical role in vasodilation under conditions of increased blood flow (Landmesser et al., 2004). During increased flow, ROS are also produced by endothelial nitric oxide synthase (eNOS) but are usually scavenged by vascular antioxidant enzymes and, subsequently, NO diffuses into adjacent smooth muscle cells and mediates vasodilation (Wolin, 2000).

Repetitive increases in blood flow during chronic exercise training have been shown to improve NO-mediated vasodilation as assessed by FMD (Walsh et al., 2003; Olson et al., 2006) and may protect against vascular dysfunction through regulation of eNOS and reduction of oxidative stress (Hambrecht et al., 2003). However, acute exercise induces oxidative stress in individuals who are unaccustomed to exercise training by enhancing vascular ROS production, which contributes to a decrease in NO bioavailability and impairs FMD. Similar to previous studies (Phillips et al., 2011), brachial artery FMD after a single bout of weight lifting was reduced in sedentary adults (Table 2). Since a sedentary lifestyle is closely linked to obesity and both are associated with vascular dysfunction and increased risk of CVD, we hypothesized the degree of FMD impairment to be augmented in OB compared to LN adults. Although FMD was reduced in sedentary OB adults after SWL, the absolute difference (pre- vs. post-SWL) was similar between groups. Other studies have found impaired FMD responses after acute aerobic exercise in sedentary overweight and obese men (Harris et al., 2008). Other studies found reduced FMD following hand grip exercise in individuals at risk of CVD (McGowan et al., 2006). To our knowledge, this is the first study to determine the effects of acute resistance exercise on FMD responses in obese women.

Previous studies showed that vascular smooth muscle responses to NTG are impaired in the presence of obesity (Ayer et al., 2011) and other CVD risk factors (Raitakari et al., 2004; Adams et al., 2005); however, other studies examining NTG-dilations in obesity independent of other risk factors suggest that it is preserved. Of considerable interest were the findings in this study that brachial artery EID responses to NTG were lower in OB compared to LN women (Figure 3). This result may represent a mechanistically different effect of exercise on FMD whereby vascular sensitivity to NO may contribute to reduce FMD in obesity compared to lean adults. Moreover, NTG-induced dilation after acute resistance exercise was associated with body weight, BMI, and WC with a tendency to correlate with %BF but not the other metabolic risk factors assessed (i.e., total cholesterol and LDL cholesterol). Although SBP and DBP were higher in OB women, there was no relationship between baseline values and NTG-induced vasodilation. Taken together, these data suggest an independent link between obesity and reduced vascular smooth muscle function after a single bout of SWL.

Both inflammation and oxidative stress could influence EID by reducing bioconversion of NTG to or scavenging of NO in smooth muscle (Schulz et al., 2002). In fact, obesity is associated with a pro-inflammatory milieu characterized by increased production of pro-atherogenic adipokines that enhance production of pro-inflammatory cytokines and increase ROS generation promoting oxidative stress (Tilg and Moschen, 2006). Acute resistance and aerobic exercise has been shown to increase plasma markers of oxidative stress in obesity (Vincent et al., 2004). Future studies will focus on the specific vascular effects of acute resistance exercise and other physical stressors on smooth muscle sensitivity to NO during obesity.

### Study limitations

There are some limitations of this study. First, the generalizability of the study is a limited to flow mediated dilation responses in women up to an hour following acute exertion. However, given that the burden of CVD is increasing in women, the results of this study contribute to a better understanding of the mechanisms by which exercise influences vascular health in women. Sex specific influences of physical stress on endothelium-dependent and endothelium-independent vasodilator responses may be important considerations in the future. Second, we were unable to evaluate the effects of NTG in sedentary OB and LN adults before a single bout of SWL due to the residual vasodilator effects of NTG on blood pressure during exercise. However, we have found that NTG dilations in a similar cohort of obese subjects were similar before and after exercise (Mean: 21% ± 1.4) suggesting that lower NTG dilation in obese women is not mediated by weight lifting. Third, CVD risk factors may have influenced NTG-dilations after SWL in OB adults since baseline blood pressure, total cholesterol, and LDL levels were higher compared to LN adults. However, in our analysis there were no relationships between these CVD risk factors and EID after acute resistance exercise. Finally, our results may have been confounded by nutritional variation and that the % body fat in the lean group was 27% a level that may be associated with metabolic dysfunction. There was no relationship between % body fat and FMD.

### Clinical insights and conclusions

In conclusion, our results suggest that acute physical stress induced by resistance exercise reduces FMD in obese women who are sedentary. Atherosclerotic CVD is a leading cause of preventable death among women between the ages of 25 and 44 and a large majority of women with CVD are asymptomatic (Maas et al., 2011) which makes early detection difficult. Since FMD correlates well with coronary artery function (Hashimoto et al., 2000) the results of our studies may extend to predict CVD risk among young women since acute exertion often triggers myocardial infarction in individuals who are sedentary (Mittleman et al., 1993).

In addition, endothelium-independent dilation to nitroglycerin was reduced in sedentary obese compared to lean women suggesting changes in vasodilator sensitivity of blood vessels to NO in obese adults. Reduced arterial function (FMD) and reduced sensitivity to NO donors (NTG) after acute physical exertion may be an important composite marker for future CV risk in young obese women without baseline arterial dysfunction or overt disease. Furthermore, these findings may be important in understanding the link between obesity and vascular dysfunction and may have important implications for how vascular and hemodynamic responses to other acute physiological perturbations such as acute hypertension, systemic hypoxemia, and hyperglycemia are altered during obesity.

### Conflict of interest statement

The authors declare that the research was conducted in the absence of any commercial or financial relationships that could be construed as a potential conflict of interest.
